# Supplementary figures and images for: A nanobody toolbox to investigate localisation and dynamics of Drosophila titins and other key sarcomeric proteins
Source: eLife. 2023 Jan 16;12:e79343. doi: 10.7554/eLife.79343 (PMC9886281; doi:10.7554/eLife.79343)

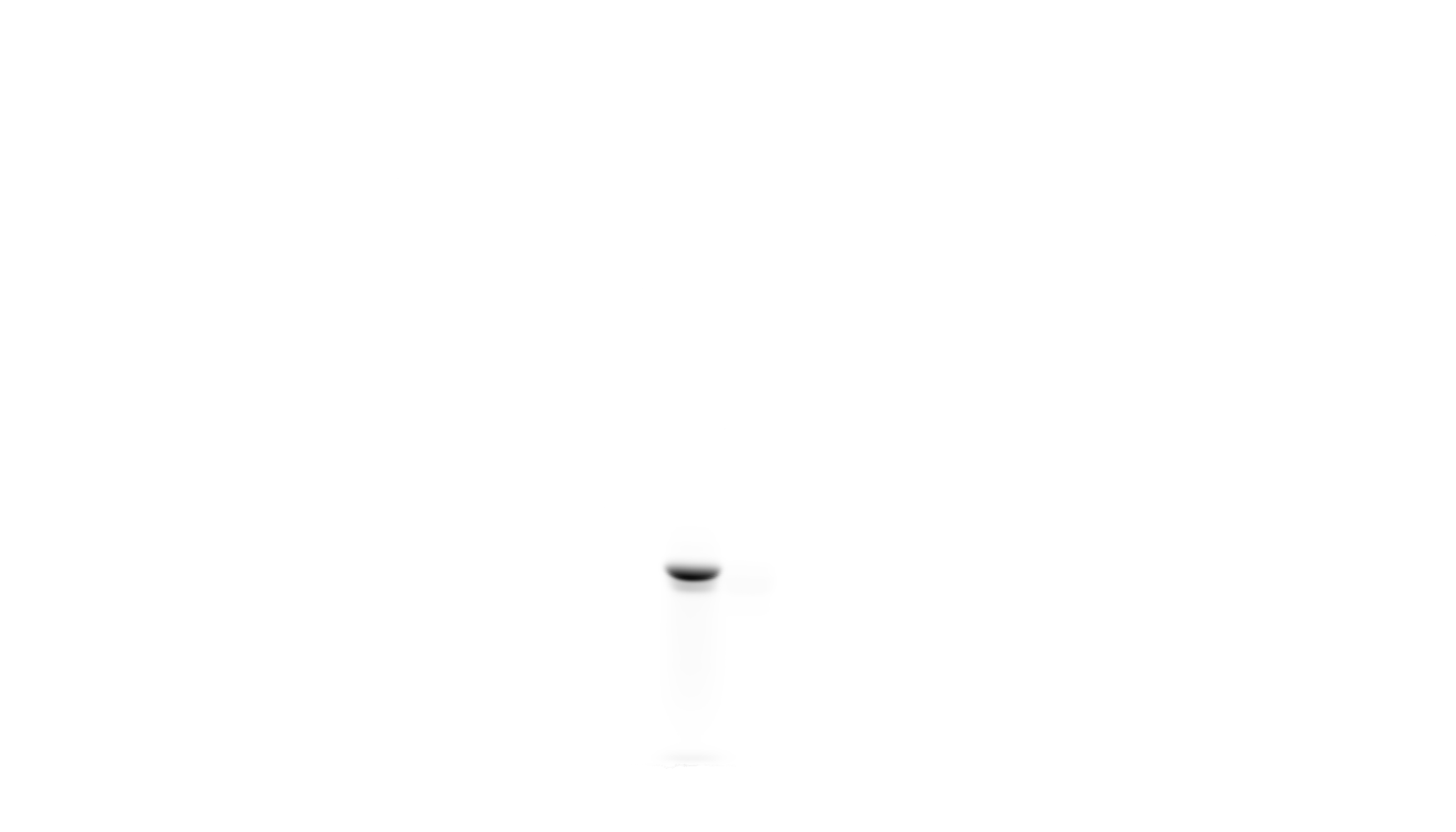

Supplement: Figure 3—source data 1. [file elife-79343-fig3-data1.zip › Figure3A_479nm.tif]

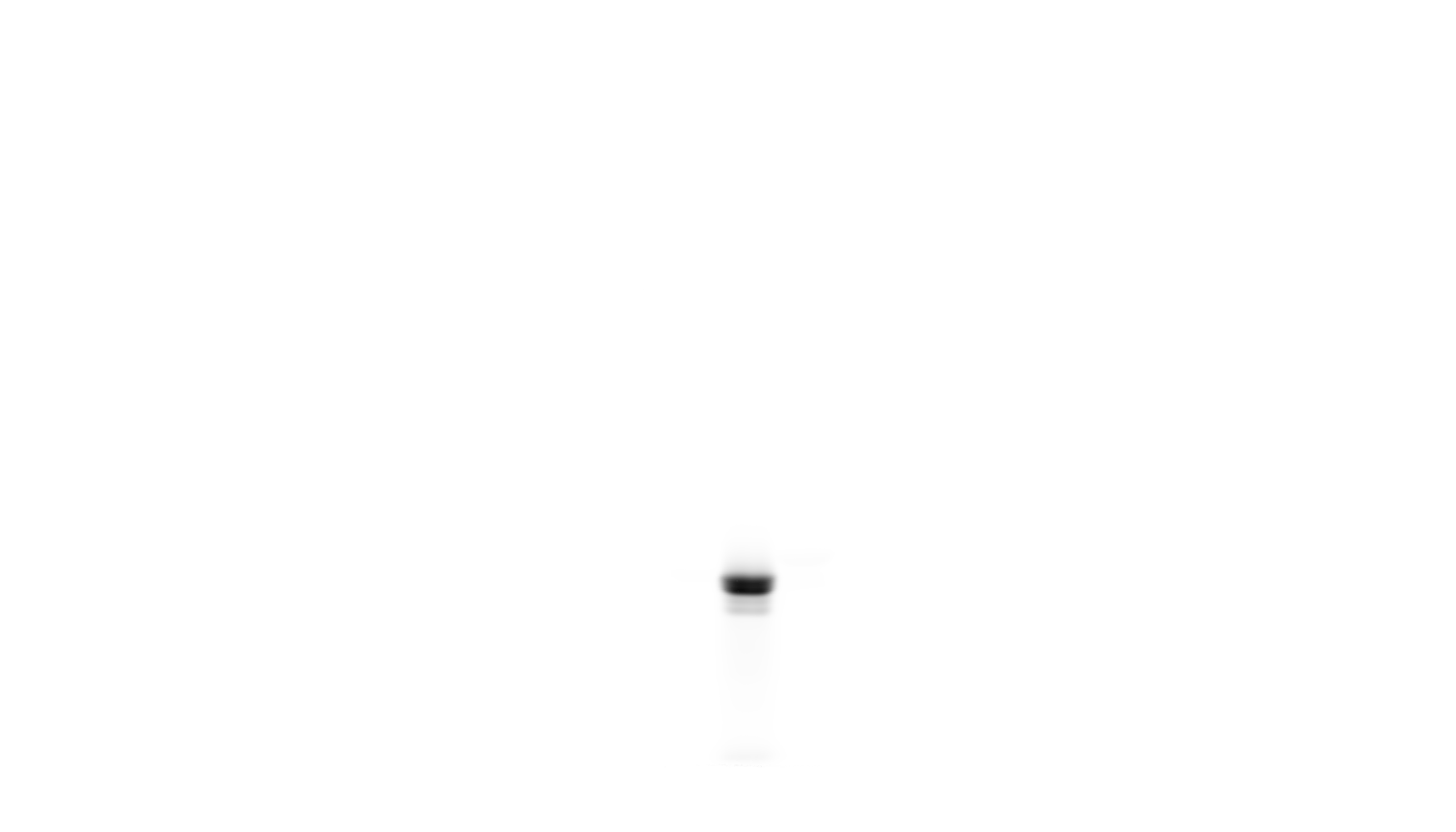

Supplement: Figure 3—source data 1. [file elife-79343-fig3-data1.zip › Figure3A_532nm.tif]

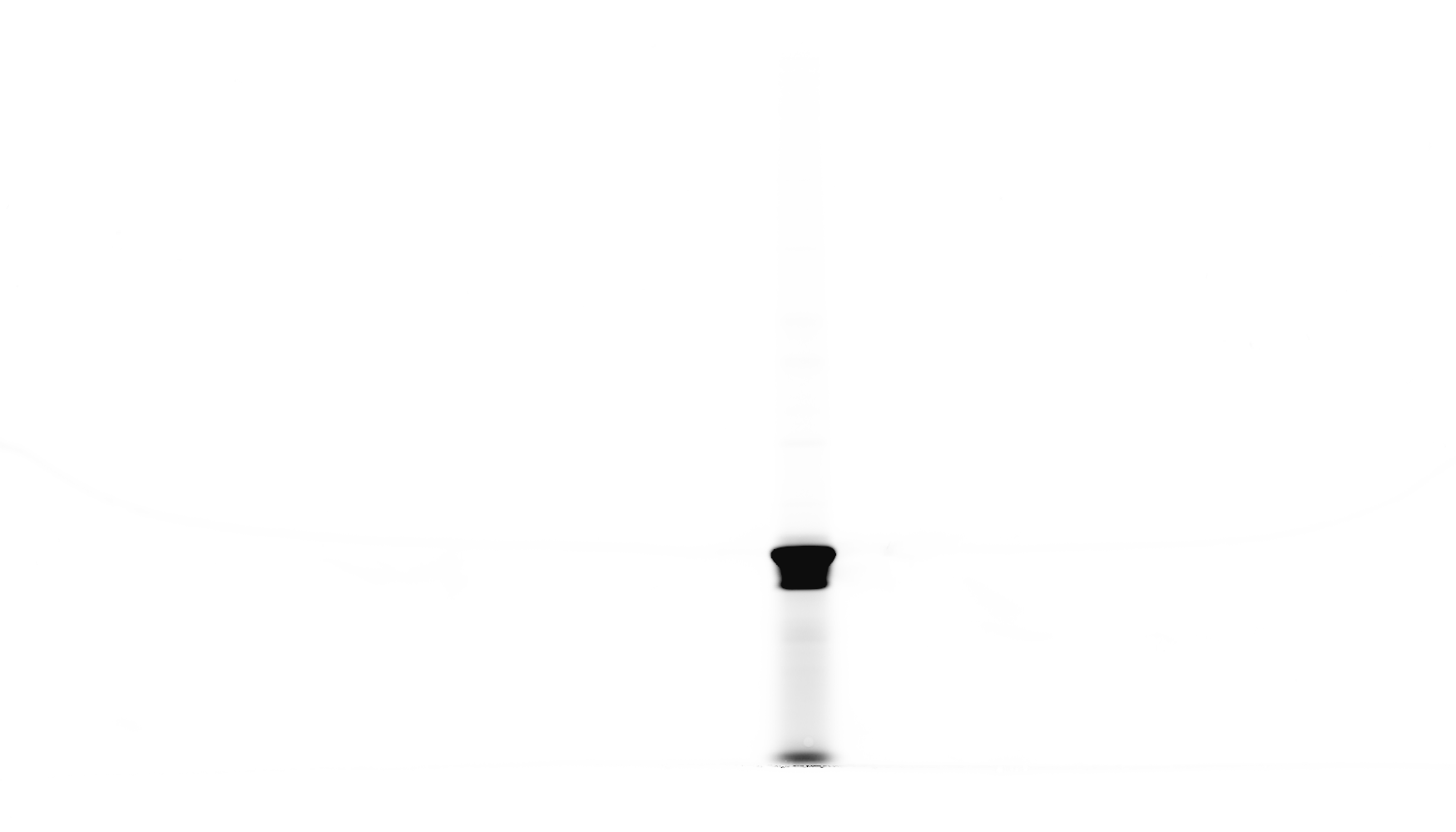

Supplement: Figure 3—source data 1. [file elife-79343-fig3-data1.zip › Figure3A_685nm.tif]

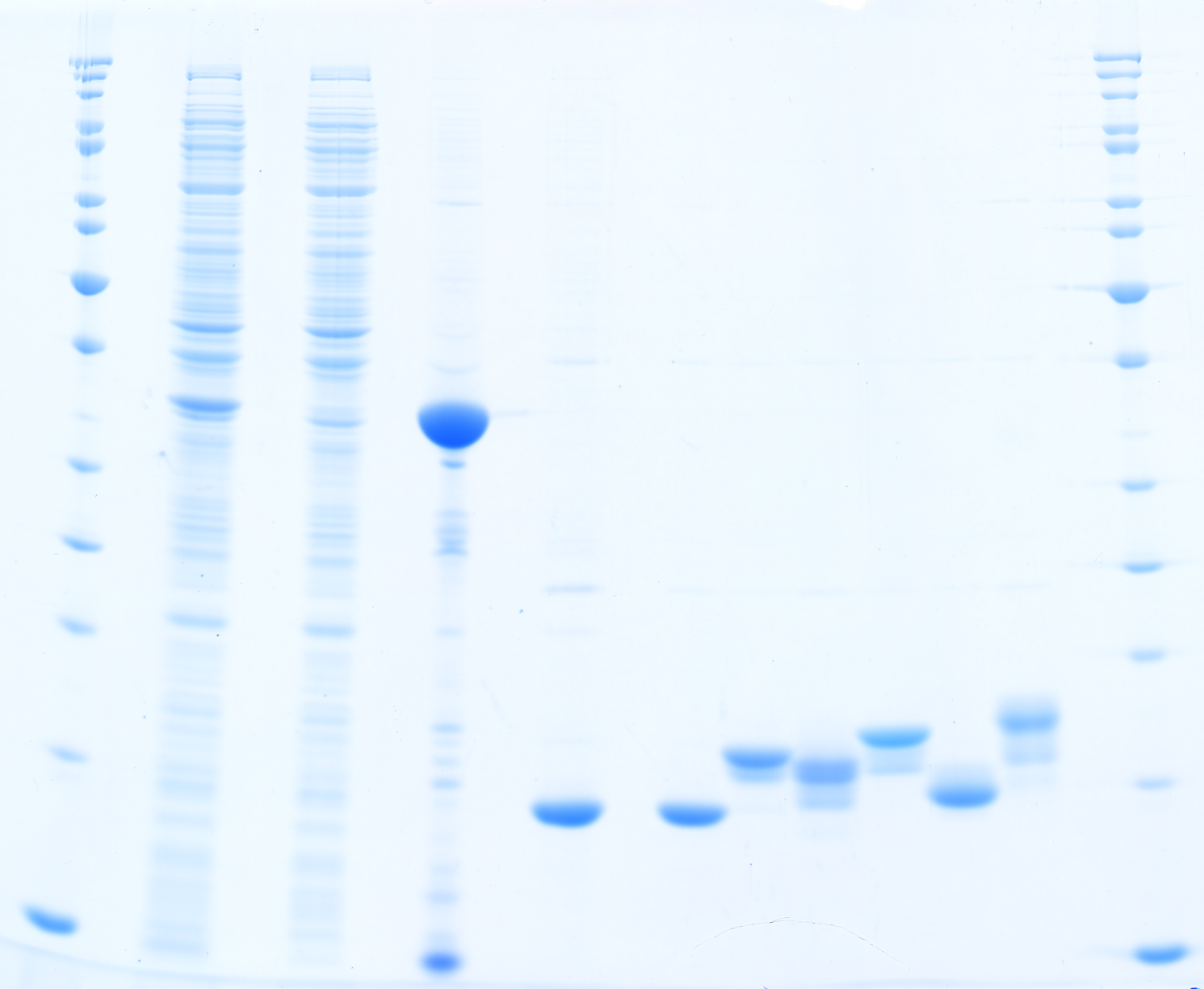

Supplement: Figure 3—source data 1. [file elife-79343-fig3-data1.zip › Figure3A_Coomassie.tif]

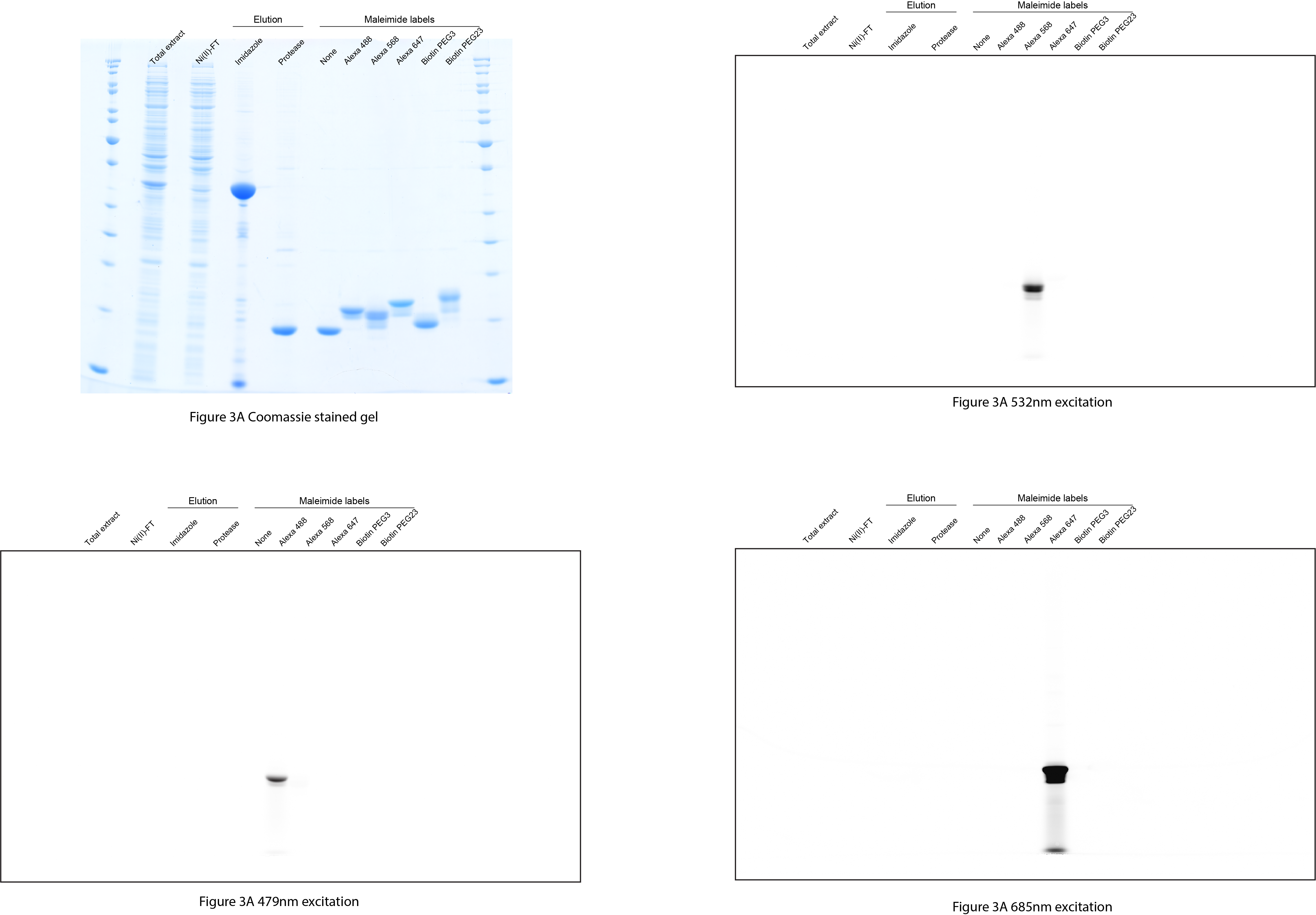

Supplement: Figure 3—source data 1. [file elife-79343-fig3-data1.zip › Figure3A_gels_labelled.png]
